# Uncoupling Foam Fractionation and Foam Adsorption for Enhanced Biosurfactant Synthesis and Recovery

**DOI:** 10.3390/microorganisms8122029

**Published:** 2020-12-18

**Authors:** Christian C. Blesken, Tessa Strümpfler, Till Tiso, Lars M. Blank

**Affiliations:** iAMB—Institute of Applied Microbiology, ABBt—Aachen Biology and Biotechnology, RWTH Aachen University, 52074 Aachen, Germany; carl.blesken@rwth-aachen.de (C.C.B.); tessa.struempfler@rwth-aachen.de (T.S.)

**Keywords:** biosurfactant, rhamnolipid, 3-(3-hydroxyalkanoyloxy)alkanoic acid (HAA), integrated product recovery, foam fractionation, foam adsorption, scale-up, metabolic engineering

## Abstract

The production of biosurfactants is often hampered by excessive foaming in the bioreactor, impacting system scale-up and downstream processing. Foam fractionation was proposed to tackle this challenge by combining in situ product removal with a pre-purification step. In previous studies, foam fractionation was coupled to bioreactor operation, hence it was operated at suboptimal parameters. Here, we use an external fractionation column to decouple biosurfactant production from foam fractionation, enabling continuous surfactant separation, which is especially suited for system scale-up. As a subsequent product recovery step, continuous foam adsorption was integrated into the process. The configuration is evaluated for rhamnolipid (RL) or 3-(3-hydroxyalkanoyloxy)alkanoic acid (HAA, i.e., RL precursor) production by recombinant non-pathogenic *Pseudomonas putida* KT2440. Surfactant concentrations of 7.5 g_RL_/L and 2.0 g_HAA_/L were obtained in the fractionated foam. 4.7 g RLs and 2.8 g HAAs could be separated in the 2-stage recovery process within 36 h from a 2 L culture volume. With a culture volume scale-up to 9 L, 16 g RLs were adsorbed, and the space-time yield (STY) increased by 31% to ${0.21\;g}_{\mathrm{RL}}/L\cdot h$. We demonstrate a well-performing process design for biosurfactant production and recovery as a contribution to a vital bioeconomy.

## 1. Introduction

Biosurfactants are microbiologically synthesized amphiphilic, surface-active substances. The hydrophilic moiety of these surfactants consists of an ester, hydroxyl, phosphate, or carboxyl group, or of carbohydrates, peptides, or proteins. The hydrophobic moiety is formed by saturated or unsaturated fatty acids, hydroxy fatty acids, or fatty alcohols [1]. Based on their abilities to lower the surface tension, increase solubility, wetting ability, and foaming capacity, surfactants are used industrially as adhesives, flocculating agents, deemulsifiers, and penetrants [2,3]. Moreover, biosurfactants demonstrate environmentally friendly properties such as production from renewable carbon sources, complete biodegradability, and low ecotoxicity [4]. Among biosurfactants, glycolipids show an especially high relevance for a broad range of industrial applications [5,6]. The industrial production of glycolipids started with sophorolipids in the last decade by several companies [7,8]. Besides sophorolipids, rhamnolipids (RLs) are the most studied glycolipids with industrial potential [1,9,10,11,12], as they, e.g., can be produced at titers above 35 g_RL_/L [13,14]. RLs consist of one or two rhamnose molecules, linked through a β-glycosidic bond to one or two 3-hydroxy fatty acid moieties [15,16]. The two fatty acids are linked by the 3-hydroxyacyl-ACP:3-hydroxyacyl-ACP O-3-hydroxy-acyl-transferase (RhlA), producing 3-(3-hydroxyalkanoyloxy)alkanoic acid (HAA). The following glycosidic bond to rhamnose is fused by the rhamnosyltransferase I (RhlB) [17]. Both products, mono-RLs and HAAs are secreted by the producing bacteria. The native and predominant producer is the opportunistic pathogen *Pseudomonas aeruginosa* [18,19]. To reduce production costs and prevent health concerns, efforts to develop a competitive non-pathogenic RL and HAA production host have increased significantly over the last two decades [12,20,21,22]. In this study, we use *Pseudomonas putida* KT2440 strains with a corresponding integration of the *rhlA* and *rhlB* genes as constructed previously [23,24,25].

Recently, Evonik Industries AG (Essen, Germany) reported a first large scale RL production using *P. putida* [26]. Even though not much is known about the process developed by Evonik, the control of excessive foaming was declared as a major challenge, agreeing with others who have discussed large scale RL production [27,28,29]. Foaming occurs when air is introduced via bubbles into the culture broth of aerobic bacteria, secreting biosurfactants. The secreted RLs and HAAs adsorb with their hydrophobic moiety, i.e., the hydrocarbon chains of the fatty acids, onto the gas-liquid interface in between the rising gas bubbles and the culture medium. When the thereby stabilized gas bubbles reach the reactor headspace, interstitial liquid is entrained by the hydrophilic part of the surfactants between the bubbles, forming the foam lamellae [30,31]. Consequently, the cultivation process is hampered, e.g., by loss of medium and bacterial cells entrapped in the foam, by a reduced oxygen transfer from the headspace into the culture, and a generally increased system heterogeneity [32,33].

Published approaches to prevent or destroy foam in a RL production process can be classified in physicochemical and mechanical techniques. Physicochemically, foam reduction in an aerated reactor is achieved by a lowered pH value or an organic phase [34,35,36,37,38]. Organic solvents are used as antifoam detergents or as an extraction agent. A reduced biocatalytic efficiency must be considered when organisms are stressed by an unfavored pH or the presence of solvents. Even though *P. putida* is known for a broad solvent tolerance [39], additional agents as antifoam generally lead to higher production costs and a more complex downstream processing (DSP) [40,41]. In this context, an in situ extraction with a biocompatible and low-priced solvent followed by solvent recycling was recently presented by Demling et al. [42]. Proven mechanical systems to prevent or limit surfactant mediated foaming are specific gassing membranes for bubble-free aeration or foam destruction by compression or centrifugation [14,40,43,44,45,46]. Such installations cannot avoid inhomogeneities in the reactor headspace and external pipelines. Here, again, *P. putida* stands out as a robust microbial cell factory, e.g., capable of enduring glucose limitations and temperature variations [47,48].

**Figure 1:**
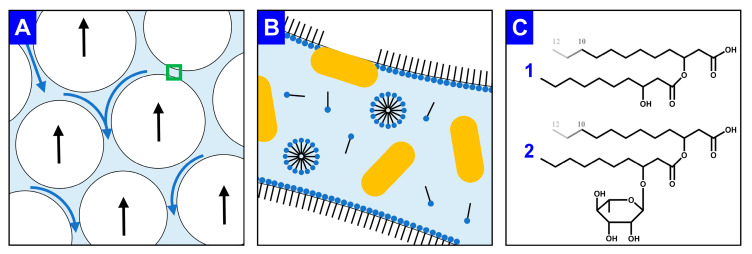
Graphical explanation of foam fractionation and foam stability as well as the molecular structures of RLs and HAAs. (**A**) Rising gas bubbles (white, black arrows indicate flow directions) and draining interstitial liquid (light blue, blue arrows indicate flow directions). The foam lamella marked with a green frame is enlarged in (**B**) with surfactants (black line: hydrophobic moiety; blue points: hydrophilic moiety) either adsorbed on the gas-liquid interface, agglomerated as micelles, or dissolved in the liquid. Pseudomonads (yellow) are suspended in the liquid or adsorbed on the gas-liquid interphase by hydrophobic cell surface structures. The molecular structure of the produced surfactants is shown in (**C**), for (**1**) HAA and (**2**) mono-RL, considering that the hydrocarbon chain length varies between C_8_ and C_12_ for the applied whole-cell biocatalysts.

In an alternative approach, the secreted surfactants are permanently separated from the ongoing cultivation process, leading not only to a lowered foaming of the culture broth but also to a product enrichment, i.e., a first DSP step. A promising technique is to separate surface-active product from the liquid culture through the foam that is already highly enriched in the surfactant. The so-called foam fractionation is considered a cost-effective and simple purification step for surfactants [41,49]. As the costs of the DSP generally account for the largest share of the entire biosurfactant production costs [18], an integrated foam fractionation can be particularly beneficial to reduce production costs. In the rising foam, gravity drainage of the interstitial liquid leads to the thinning of the foam lamella (Figure 1A) [31,50]. With surfactants adsorbing onto the gas-liquid interface to decrease the Gibbs free energy, the gas bubbles gain higher stability (Figure 1B) [51]. Due to hydrophobic surface structures, bacterial cells also adsorb on the interface. To reduce biomass content in the foam, engineered *P. putida* strains with genetic deletions of such hydrophobic surface structures were recently reported [24]. Briefly, bacterial cells lacking the flagellum or the large adhesion protein F agglomerate in the foam to a lower extent than *P. putida* KT2440 without surface modifications. Solved surfactants and suspended cells drain through the foam lamella. Furthermore, micelle formation for mono-RLs and HAAs in the interstitial liquid occurs at about 0.1 g/L [38,52]. The molecular structures reveal the amphiphilic character of the applied surfactants, i.e., HAAs and RLs (Figure 1C). Next to the hydrophobic hydrocarbon chains, the carboxyl and hydroxy group of the HAAs and the additional rhamnose molecule of the mono-RLs are hydrophilic. Therefore, not only RLs but also the aglyconic HAAs are considered as biosurfactants [53].

Foam fractionation is known as a cost-effective and efficient technology for the separation of biosurfactants as RLs, surfactins, pseudofactins, and hydrophobin proteins from a culture broth [30,54,55,56,57,58]. An overview of integrated RL separations via discharged foam directly from the reactor headspace is given in Table 1. First comprehensive works in this field were performed at the Department of Biotechnology at the Technical University of Braunschweig in the 1980s, summarized by Siemann et al. [59]. *P. aeruginosa* was immobilized to prevent loss of the whole-cell biocatalyst from being entrapped in the discharged foam. With the introduction of non-pathogenic RL producer strains by Wittgens et al. [22], the development of scalable bioreactor processes was enforced in the last five years by Beuker et al. [60], Anic et al. [61], and Blesken et al. [24].

Although these works report successful setups and performances, two major challenges are paramount. First, the bacterial foam adhesion causes loss of biocatalyst in the bioreactor. Second, process parameters as bioreactor stirring and gassing rates as well as headspace and fractionation column dimensions have a major impact on the fractionation performance.

Here we present a novel bioreactor setup with an integrated but independently operated foam fractionation column. Such a system allows a higher degree of freedom to adjust the process parameters in both unit operations. In comparison to previously published setups, not the reactor headspace, but an external vertical column is intended to be the central element for foam fractionation. This technique promotes a decoupling of the fractionation process from the cultivation process. Furthermore, we want to achieve continuous product recovery that is particularly important to produce HAAs, which are unstable in the culture broth [25,52]. The product recovery should be performed via foam adsorption, a patented technique for RL adsorption directly from the lamellae of the foam [63]. The foam adsorption unit, as constructed by Anic et al. [61], will be connected to the upper outlet of the fractionation column. With a subsequent desorption, a product harvest can be achieved directly from the process. We want to show that less medium in the fractionated foam contributes to a lowered load of the adsorption column by microbial cells and side products. In summary, the novel setup contributes to a scalable and rather simple biosurfactant production with integrated product recovery.

## 2. Materials and Methods

### 2.1. Bacterial Strains

The applied RL and HAA production hosts *P. putida* KT2440 ∆flag_RL, and *P. putida* KT2440 ∆*lapF*_HAA were constructed as described previously [24]. Briefly, for the RL and HAA production strains, mini-Tn7 delivery transposon vectors pSK02 [64], harboring the *rhlAB* genes and pKS03 [24], harboring the *rhlA* gene, were integrated into the genome as described by Zobel et al. [65]. For this purpose, *rhlAB* genes were isolated and amplified from the pathogen *P. aeruginosa*. For specific cell surface deletions (∆flag and ∆*lapF*), the pEMG-system was used as described by Martinez-Garcia et al. [66].

### 2.2. Culture Conditions

For cultivation, cryo-cultures were spread onto lysogeny broth (LB) agar (10 g/L tryptone, 5 g/L yeast extract, 10 g/L NaCl, 20 g/L agar). After 15 h incubation at 30 °C, cells were transferred to 5 mL LB medium in a test tube and shaken at 200 rpm with a 50 mm shaking diameter at 30°C in a Multitron Pro shaker (Infors AG, Bottmingen, Switzerland). After 12 h, 50 mL minimal medium with 10 g/L glucose were inoculated at an optical density at 600 nm (OD_600_) of 0.2 for *P. putida* KT2440 ∆flag_RL and 0.4 for *P. putida* KT2440 ∆*lapF*_HAA, in a 500 mL flask. For the precultures and accordingly for the following bioreactor culture, higher initial biomass concentrations were chosen for the HAA producer to guarantee the same timing for the production of both surfactants. Pretests (not shown) confirmed that higher cell densities as the one applied for the RL producer are required for a stable foam fractionation with HAAs. The flasks were shaken for 8 h at 300 rpm in the same shaker previously used for the test tubes. To all cultures, from agar plate to shake flask, 25 mg/L gentamicin was added to prevent contamination. The applied minimal medium is based upon the mineral salt medium (MSM) by Hartmans et al. [67] with a modified phosphate buffer at pH 7. For shake flask cultivation, 11.64 g K_2_HPO_4_ and 4.89 g NaH_2_PO_4_ were used (per L). In fermenters, 3.88 g K_2_HPO_4_, and 1.63 g NaH_2_PO_4_ were applied (per L) and the pH was adjusted via 30% (*v*/*v*) NH_4_OH. Further medium components were (per L) 2 g (NH_4_)_2_SO_4_ and the trace elements 10 mg EDTA, 0.1 mg MgCl_2_∙6H_2_O, 2 mg ZnSO_4_∙7H_2_O, 1 mg CaCl_2_∙2H_2_O, 5 mg FeSO_4_∙7H_2_O, 0.2 mg Na_2_MoO_4_∙2H_2_O, 0.2 mg CuSO_4_∙5H_2_O, 0.4 mg CoCl_2_∙6H_2_O, and 1 mg MnCl_2_∙2H_2_O.

### 2.3. Fermentation Setup and Procedure

#### 2.3.1. Setup and Procedure for 2 L Scale

**Figure 2:**
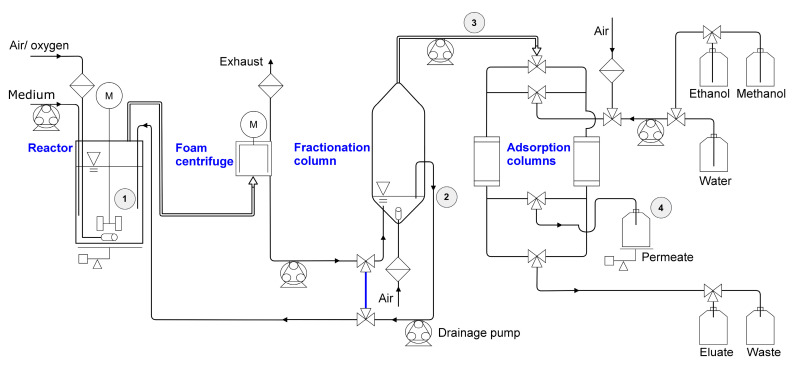
Fermentation setup with the two stages in the growth phase, and the harvest phase. Growth phase: 1st stage: no gassing into the stirred bioreactor. 2nd stage: activated gassing; discharging foam through the exhaust into the foam centrifuge; foamate reflux into the reactor (bypass in blue). Harvest phase: introduced by stopping the reflux and guiding the foamate into the fractionation column, equipped with an aeration and a separation of drained liquid back into the bioreactor. Fractionated foam left the upper opening of the fractionation column into the automated adsorption unit with two alternating adsorption columns. Permeate was collected and weighed, the eluate was collected separately. Bioreactor working volume was maintained by weight-controlled refill. Sampling points are marked as ① reactor, ② drainage reflux, ③ fractionated foam, ④ permeate inlet.

The fermentation was performed using a BioFlo 120 bioreactor system with the DASware control (Version 5.0) software package (both from Eppendorf AG, Hamburg, Germany) in combination with two external peristaltic pumps for medium supply (120U, Watson-Marlow Limited, Falmouth, UK). The conducted fermentation procedure was separated into two phases, the growth phase to gain a certain biomass concentration and the following harvest phase. The complete setup is illustrated in Figure 2, and a picture is provided as Supplementary Figure S1. In the first stage of the growth phase, 1.5 L minimal medium, including 10 g/L glucose, was inoculated with a *P. putida* KT2440 ∆flag_RL preculture to an OD_600_ of 0.2 and with a *P. putida* KT2440 ∆*lapF*_HAA preculture to an OD_600_ of 0.4. For both cultures, the inoculated volume of shake flask preculture was always less than 6% (*v*/*v*) of the working volume.

When the *P. putida* KT2440 ∆flag_RL culture reached an OD_600_ > 0.6, and the *P. putida* KT2440 ∆*lapF*_HAA an OD_600_ > 1, the second stage of the growth phase was initiated. The gassing through a sintered sparger (bbi-biotech GmbH, Berlin, Germany) was turned on (${\dot{V}}_{\mathrm{gassing}\;}=$ 0.4 L/min; 0.27 vvm) to prevent oxygen limitation. The dissolved oxygen (DO) content was maintained at 30% by the appropriate addition of pure oxygen. The appearing foam left the reactor through the air exhaust into a foam centrifuge (Foamex 5, Heinrich Frings GmbH & Co. KG, Rheinbach, Germany). The foamate leaving the centrifuge was pumped back into the reactor (530U, Watson-Marlow Limited). 4 h after inoculation, the foamate reflux was stopped, initiating the shift from growth to harvest phase. For the harvest, the foamate was led into the bottom of the fractionation column (Ø_inner_ = 115 mm, h= 330 mm). A tube with its inlet 30 mm above the foamate inlet was inserted into the column to transport excess liquid back into the fermenter to keep the liquid level in the fractionation column constant. In the following, this removed liquid is defined as drainage reflux. To enable surfactant adsorption onto the gas-liquid interface, an air flow of 10 L/h at an overpressure of 0.5 bar was led into the pool with a sintered sparger (bbi-biotech GmbH, Berlin, Germany). At the beginning of the harvest phase, 500 mL minimal medium with three times the concentration of trace elements compared to the initial medium composition, defined as feed medium, were pumped into the reactor. In the further course of the cultivation, the liquid level in the reactor was maintained by weight control. When the liquid was removed from the system with the separated foam, a correspondent amount of feed medium was automatically pumped into the reactor. After the first 16 h of the harvest phase, to control growth, fresh medium was exchanged for 0.9% (*w*/*v*) NaCl. During the entire harvest phase, glucose was introduced by a DO-based feeding system. When the DO reached values > 55% due to an inhibited respiration caused by a lack of dissolved carbon, a 50% (*w*/*v*) glucose solution was pulsed into the broth. Additional process parameters are listed in Supplementary Table S1. The fractionated foam was led to an automated adsorption unit, initially constructed by Anic et al. [61]. The unit enabled automated adsorption, desorption, and regeneration of two packed bed adsorption columns which were loaded alternately for 8 h each. The desorption and regeneration followed a specific sequence in which either liquid was driven out of the column by air, the packed bed was flushed with distilled water, or the product was eluted with ethanol or methanol. A detailed order of the applied desorption and regeneration steps is given in Supplementary Table S2. The eluate was collected separately. During adsorption, surfactant-free permeate was collected and weighed. The designed adsorption columns (Ø_inner_ = 59 mm) had an adjustable adapter to obtain a compressed packed bed. As adsorption material, 30 g of hydrophobic C_18_ silica-based ODS-A (Octadecylsilyl-A AA12SA5, pore size: 12 nm, particle size: 150 μm; YMC Co., Ltd., Kyoto, Japan) was applied for each column, resulting in a packed bed volume of about 56 cm^3^. The fermentation was terminated after 4 adsorption cycles, resulting in a 32 h harvest phase and 36 h of total fermentation time.

#### 2.3.2. Setup and Procedure for 9 L Scale

A fermentation process with integrated foam fractionation and adsorption was performed with 9 L minimal medium, including 20 g/L glucose, with the RL producer *P. putida* KT2440 ∆flag_RL. Apart from the bioreactor vessel (also from Eppendorf AG), the fermentation setup was the same as for the 2 L scale (see Section 2.3.1). The cultivation and product separation process changes are described in the following. The reactor medium was inoculated with a preculture (<2% (*v*/*v*) of the working volume) to an OD_600_ = 0.05. When the culture reached an OD_600_ > 0.6, the reactor gassing was turned on (${\dot{V}}_{\mathrm{air}}\;=$ 1 L/min; 0.125 vvm) to maintain a DO value ≥ 30%. The harvest phase started after 12 h when the culture reached an OD_600_ = 13. The culture was supplemented with 1 L feed medium, resulting in a reduced gassing of 0.11 vvm. Further process parameters are listed in Supplementary Table S3. As for the 2 L scale, oxygen limitation was prevented by an appropriate addition of pure oxygen. The fractionated foam was led to the adsorption column with a packed bed of 60 g of hydrophobic C_18_ silica-based ODS-A. The adsorption column load alternated every 8.5 h. For RL desorption, only ethanol was used as eluent during the alternating adsorption/ desorption procedure. Methanol was applied for a final elution after the last adsorption for each column. A detailed order of the applied desorption and regeneration steps is given in Supplementary Table S4. After the first 12.5 h of the harvest phase, the feed medium was already replaced with 0.9% (*w*/*v*) NaCl to reduce the synthesis of further RLs but continuing the air stripping of the already accumulated surfactants from the reactor.

#### 2.3.3. Determination of HAA Adsorption Capacity

For the determination of the maximum adsorption of HAAs by the applied C_18_ silica-based ODS-A material, HAAs were purified via preparative HPLC, according to a modified RL purification method from Blesken et al. [24]. For HAA purification, the elution gradient of the method was maintained at 100% acetonitrile until a retention time of 55 min. HAAs were fractionated in between 33 min and 38 min retention time. The HAAs were dissolved in ultrapure water and the solution was set to a neutral pH via 0.5 M HCl and 1 M NaOH. 14 mL HAA solution with the concentrations 2.06 g/L, 1.65 g/L, 1.4 g/L, 1.2 g/L, 0.97 g/L, 0.58 g/L, 0.34 g/L, and 0.18 g/L were filled in 15 mL reaction tubes and supplemented with 13 mg adsorbent (i.e., a surface of 5 m^2^). After mixing for 2 h at 20 rpm with a Stuart SB2 Rotator (Barloworld Scientific Ltd., Staffordshire, UK) at 30 °C, samples were taken from the supernatant for determination of the HAA concentration.

### 2.4. Sampling and Analytics

Samples were taken from the reactor broth, the drainage reflux, the fractionated foam, and the permeate inlet into the collecting bottle (Figure 2). The foam was completely destabilized to prevent incorrect measurement by gas inclusion. The OD_600_ was measured using an Ultrospec 10 cell density meter (Biochrom, Cambridge, UK). An OD_600_ of 1.0 corresponds with a determined cell dry weight of 0.31 g_CDW, *P. putida* KT2440 ∆flag_RL_/L and 0.32 g_CDW, *P. putida* KT2440 ∆*lapF*_HAA_/L. Glucose was analyzed as described previously [68] in a Dionex Ultimate 3000 HPLC system, composed of the pump ISO-3100, the autosampler WPS-3000, and the column oven TCC-3000, connected to a DIONEX UltiMate 3000 Variable Wavelength Detector set to 210 nm (Thermo Fisher Scientific Inc., Waltham, MA, USA) and a RI detector SHODEX RI-101 (Showa Denko Europe GmbH, Munich, Germany) equipped with an ISERA Metab AAC 300 × 7.8 mm column (particle size: 10 µm, ISERA GmbH, Düren, Germany). For the determination of RL and HAA concentrations, analytical methods and sample preparations were performed according to Bator et al. [64], based on a method developed previously [69,70]. Briefly, a RP-HPLC Ultimate 3000 HPLC system, composed of the pump LPG-3400, the autosampler WPS-3000, and the column oven TCC-3000, connected to a Corona Veo charged aerosol detector (CAD) (all Thermo Fisher Scientific Inc.) equipped with a NUCLEODUR C18 Gravity 150 × 4.6 mm column (particle size: 3 µm, Macherey-Nagel GmbH & Co. KG, Düren, Germany) was used. All components were identified via the retention time and quantified via the peak area compared to corresponding standards.

### 2.5. Data Analysis

To define process parameters, the following variables are used: *V* is volume, $\dot{V}$ is volume flow, *m* is mass, $\dot{m}$ is mass flow and *c* is concentration. The index P is product, S is glucose, and X is biomass. Relevant times are the starting time *t_0_*, the sampling time *t_i_*, and the time at the end of the cultivation *t*_final_. Biomass and surfactant enrichment factors *E* are defined for each sampling point as the relevant concentration in the fractionated foam (*ff*) divided by the concentration in the reactor (Equations (1) and (2)):

(1) $$E_{{X,\;t}_{i}}\;=\;\frac{{\mathrm{OD}}_{{ff,\;t}_{i}}}{{\mathrm{OD}}_{{\mathrm{reactor},\;t}_{i}}}\;$$

(2) $$E_{{P,\;t}_{i}}\;=\;\frac{c_{{P,\;ff,\;t}_{i}}}{c_{{P,\;\mathrm{reactor},\;t}_{i}}}$$

Separated product via fractionated foam is determined for every 2 h at sampling time *t_i_* (Equations (3) and (4)):

(3) $${\dot{m}}_{{P,\;ff,\;t}_{i}}=\;\frac{\Delta m_{{P,\;ff,\;t}_{i}\;}}{2\;h}\;=\;\frac{{\bar{c}}_{{P,\;ff,\;t}_{i}}{\;(V}_{{\mathrm{permeate},\;t}_{i}\;}{-V}_{{\mathrm{permeate},\;t}_{i-2h}})}{2\;h}\;\;\;\;\;\;\;\;\;\;{\;[g}_{P,ff}/h]$$

(4) $${\bar{c}}_{{P,\;ff,\;t}_{i}\;}=\;{0.5\;(c}_{{P,\;ff,\;t}_{i}}{+\;c}_{{P,\;ff,\;t}_{i-2h}}){\;\;\;\;\;\;\;\;[g}_{P,ff}/L]$$

Surfactant recovery *R*_P_ by foam fractionation is calculated for every 2 h at sampling time *t_i_*, by the Equations (5)–(7). The quantity of product entering the fractionation column in a 2 h interval ($\;\Delta m_{P,\;\mathrm{inlet}}$) is determined by the sum of the product quantities leaving the column ($\;\Delta m_{P,\;ff\;}\&\;\Delta m_{P,\;\mathrm{drainage}\;}$).

(5) $$\begin{array}{c} R_{{P,\;t}_{i}}=\frac{\Delta m_{{P,\;ff,\;t}_{i}\;}}{\Delta m_{{P,\;\mathrm{inlet},\;t}_{i}}}\cdot 100\\ =\frac{\Delta m_{{P,\;ff,\;t}_{i}}}{\Delta m_{{P,\;ff,\;t}_{i}\;}+\;\Delta m_{{P,\;\mathrm{drainage},\;t}_{i}}}\cdot 100\;=\frac{\Delta m_{{P,\;ff,\;t}_{i}}}{\Delta m_{{P,\;ff,\;t}_{i}}{+\;\bar{c}}_{{P,\;\mathrm{drainage},\;t}_{i}\;}\;\cdot {\bar{\dot{V}}}_{{\mathrm{drainage},\;t}_{i}\;}\cdot 2\;h}\cdot 100\;\left[\%\right] \end{array}$$

(6) $${\overset{ˉ}{c}}_{P,\mathrm{drainage},t_{i}}=0.5\left(c_{P,\mathrm{drainage},t_{i}}+c_{P,\mathrm{drainage},t_{i-2h}}\right)\;\;\;\;\;\;\;\;\;\;\left[g_{P,\mathrm{drainage}}/L\right]$$

(7) $${\bar{\dot{V}}}_{{\mathrm{drainage},\;t}_{i}\;}=\;{0.5\;(\dot{V}}_{{\mathrm{drainage},\;t}_{i}}{+\dot{V}}_{{\mathrm{drainage},\;t}_{i-2h}})\;\;\;\;\;\;\left[L/h\right]$$

Surfactant separation *S*_P_ is defined as shown in Equation (8):

(8) $$S_{{P,\;t}_{\mathrm{final}}}\;=\;\frac{{\;m}_{{P,\;\mathrm{eluate},\;t}_{\mathrm{final}}}\;}{m_{{P,\;\mathrm{reactor},\;t}_{\mathrm{final}}\;}{+\;m}_{{P,\;\mathrm{eluate},\;t}_{\mathrm{final}}}}\cdot 100\;\;\;\;\;\;\left[\%\right]$$

For the final yield of product from glucose ($Y_{{P/S,\;t}_{\mathrm{final}}}$), the product separated as eluate from the process is considered (Equation (9)):

(9) $$Y_{{P/S,\;t}_{\mathrm{final}}}\;=\;\frac{m_{{P,\;\mathrm{reactor},\;t}_{\mathrm{final}}\;}{+\;m}_{{P,\;\mathrm{eluate},\;t}_{\mathrm{final}}}{-\;m}_{{P,\;\mathrm{reactor},\;t}_{0}}}{m_{{S,\;\mathrm{reactor},\;t}_{0}}{+\;m}_{S,\;\mathrm{feed}}{\;-\;m}_{{S,\;\mathrm{reactor},\;t}_{\mathrm{final}}\;}}{\;\;\;\;\;\;\;[g}_{P}{/g}_{S}]$$

The final space-time yield ${(\mathrm{STY}}_{{P,\;t}_{\mathrm{final}}})$ is defined by the absolute product quantity divided by the corresponding culture volume and cultivation time (Equation (10)):

(10) $${\mathrm{STY}}_{{P,\;t}_{\mathrm{final}}}\;=\;\frac{m_{{P,\;\mathrm{reactor},\;t}_{\mathrm{final}}\;}{+\;m}_{{P,\;\mathrm{eluate},\;t}_{\mathrm{final}}}\;{-\;m}_{{P,\;\mathrm{reactor},\;t}_{0}}}{V_{\mathrm{culture}}\cdot {\;t}_{\mathrm{final}}}{\;\;\;\;\;\;\;[g}_{P}/L\cdot h]$$

## 3. Results

### 3.1. Enhanced Fractionation Performance by Uncoupling Its Operation from Fermentation

We recently demonstrated the suitability of an integrated foam fractionation column for the continuous removal of RLs and HAAs from the bioreactor [24]. Foam fractionation was applied to maintain the biocatalysts in the fermentation broth instead of being removed from the cultivation process by bacterial foam adhesion. Simultaneously, RLs and HAAs were enriched by foam fractionation to facilitate product separation from the culture broth. However, a 2.2-fold RL enrichment and a 5-fold HAA enrichment via foam fractionation were not exceptionally high. Back then, we concluded that operating the foam fractionation via the gassing rate of the bioreactor leads to suboptimal conditions for fractionation, e.g., high gassing rates used for the appropriate oxygen supply in the culture allowed only for a short residence time of the foam in the fractionation column, leading to inefficient draining. We here developed a setup in which the foam discharged from the fermenter was first collapsed to counteract this. The foamate was then, under suitable conditions foamed out and subsequently fractionated. To avoid high biomass loss via entrained bacterial cells, we used optimized whole-cell biocatalysts previously engineered to have less hydrophobic cell surfaces.

**Figure 3:**
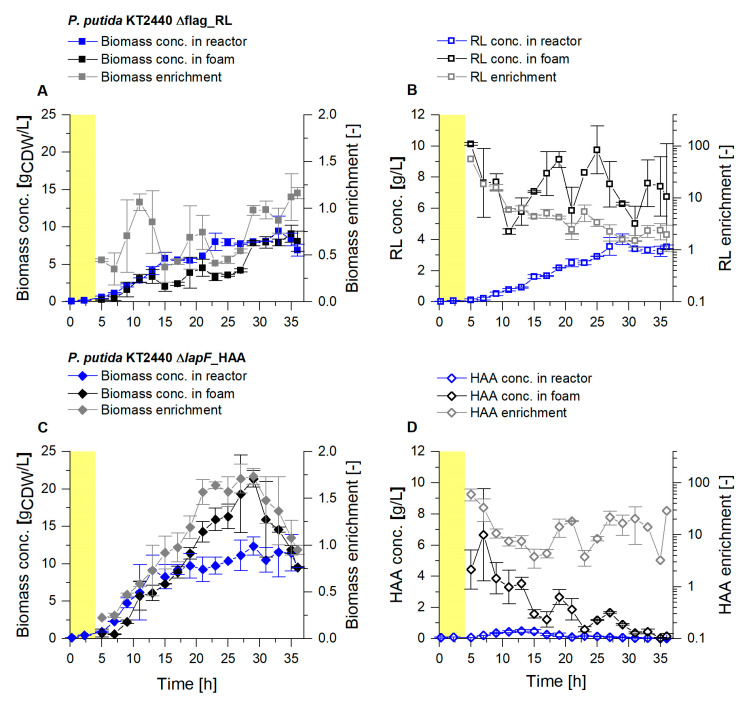
Cultivation of *P. putida* KT2440 Δflag_RL (**A**,**B**) and *P. putida* KT2440 Δ*lapF*_HAA (**C**,**D**) in a bioreactor with 2 L working volume. Growth phase (yellow background) and a subsequent continuous product separation during the harvest phase (t = 4 h to t = 36 h). (**A**,**C**) Biomass concentration in the reactor (blue) and in the fractionated foam (black) and the biomass enrichment (*E_X_*, gray). (**B**,**D**) Surfactant concentrations were measured in the fermentation broth of the reactor (blue) and in the fractionated foam (black), depicted together with the surfactant enrichment (*E_P_*, gray). The error bars indicate the deviation from the mean of two biological replicates.

In the reactor, maximal biomass concentrations of 8 g_CDW_/L for the RL producer, and above 11 g_CDW_/L for the HAA producer, were reached (Figure 3A,C). The bacterial enrichment in the foam, a crucial parameter to characterize the loss of cells by foaming, is defined by the biomass concentration in the fractionated foam, referred to the biomass concentration in the reactor ($E_{X}$, Equation (1)). Throughout the harvest phase, the average biomass enrichment was 0.7 for the RL producer. Thus a relatively low enrichment of the non-flagellated *P. putida* KT2440 Δflag_RL agrees with the previous results [24]. For the HAA producer KT2440 ∆*lapF*_HAA, an average biomass enrichment in the fractionated foam of 1.1 was determined. The enrichment of the biomass in the HAA foam constantly increased to a maximum of 1.7, with biomass concentrations above 20 g_CDW_/L in the foam, followed by a sudden drop in the last 7 h of the harvest phase. While it was intended to reach low *E_X_*-values, the enrichment of the product (*E*_P_, Equation (2)) should be preferably high. *E*_P_-values in the RL production process showed a constant decrease, caused primarily by an increasing RL concentration in the reactor while the RL concentration in the foam remained mainly at values between 6 and 9 g_RL_/L (Figure 3B). On average a 6.3-fold enrichment of RLs with the fractionated foam could be obtained. Higher initial RL concentrations caused foam with a high liquid content, which could not be reduced as achieved with lower initial concentrations in the earlier harvest phase. After 20 h, the feed medium was replaced with 0.9% (*w*/*v*) NaCl to reduce biomass growth and, consequently, lower the RL production rate. By stripping already accumulated biosurfactants out of the broth, a higher surfactant separation was envisaged ($S_{{P,\;t}_{\mathrm{final}}}$, Equation (8)). For HAA production, the enrichment of the surfactants in the foam fluctuated in a range of 3 to 60, resulting in an average enrichment of 17, a significantly higher enrichment compared to the fractionation of RL foam (Figure 3D). However, HAA concentrations in the foam were lower, with a steady decline from about 5 to below 0.5 g_HAA_/L. Even though a sufficient glucose feed was established, it is assumed that HAAs were degraded as an alternative carbon source from the pseudomonads, as described before [52].

**Figure 4:**
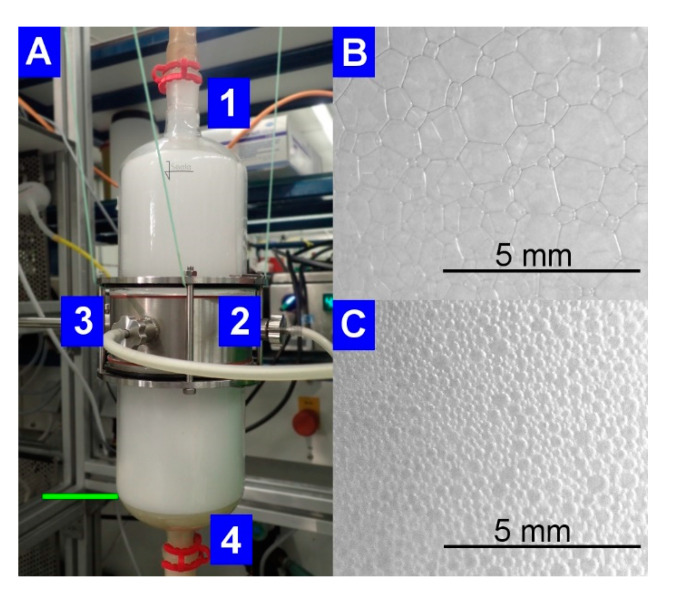
Pictures of the operating foam fractionation column at RL enrichment after 9.7 h of cultivation. (**A**) General view of the column with (**1**) the upper outlet of the fractionated foam, the connectors for (**2**) the inlet of the foamate from the bioreactor, and (**3**) the outlet for the drainage reflux and to maintain the liquid level of the pool (indicated by green line), (**4**) the sparger positioned at the column bottom. (**B**) Polyhedral foam structure at the upper outlet of the fractionation column. (**C**) Spherical foam formation just above the pool.

When observing the foam in the external foam fractionation column (Figure 4A), the decrease of the liquid content as the foam rises in the column became visible by a change of the foam structure. The spherical foam, with a high liquid content in between the gas bubbles, was formed above the pool (Figure 4C). In the upper part of the column, foam formed polyhedral structures with increased gas bubble sizes surrounded by the thin foam lamellae (Figure 4B).

**Figure 5:**
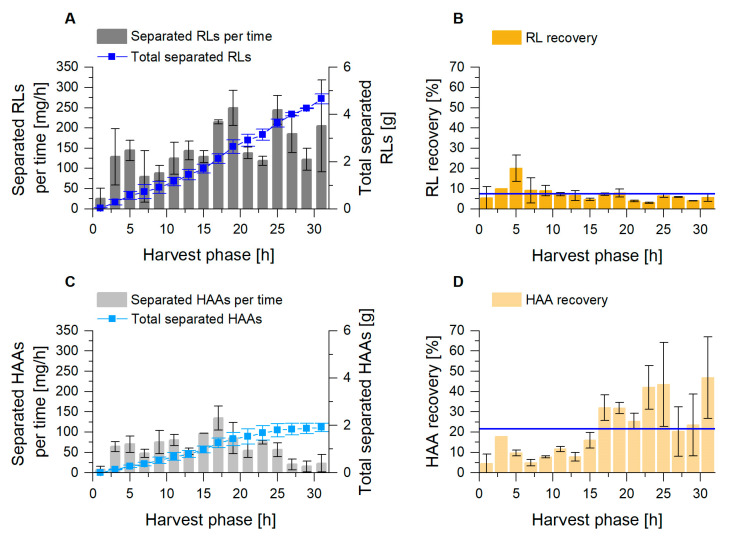
Foam fractionation performance for RL (**A**,B) and HAA (**C**,**D**) separation in the external foam fractionation column. (**A**,**C**) Separated mass flow of surfactant for every 2 h (gray columns) and the total mass of separated product (blue) during harvest phase. (**B**,**D**) Product recovery *R_P_* for every 2 h during harvest phase, with the mean value as blue line. The error bars indicate the deviation from the mean of two biological replicates.

Next to the product enrichment factors, the product quantity separated in the fractionated foam is an important parameter to assess the efficiency of the conducted fractionation. We, therefore, determined the product mass flow in the fractionated foam. The results showed a permanent product removal from the cultivation via dried foam, with average flows of 151 mg_RL_/h and 62 mg_HAA_/h (Equation (3); Figure 5A,C). The surfactant recovery *R*_P_ (Equation (5)) was defined by the amount of product in the fractionated foam related to the amount of product introduced into the fraction column for 2 h intervals. The RL foam fractionation revealed an average recovery of 7.5% (Figure 5B). The highest values were achieved in the first part of the harvest phase. For the fractionation of HAAs, an average recovery of 22% was determined, with increasing values in the second part of the harvest phase (Figure 5D). For the cultivation of *P. putida* KT2440 Δ*lapF*_HAA, the lowered HAA concentration in the broth, in combination with a consistently high *E*_P_-value in the foam, was likely decisive for product recovery values higher than 20%. In general, a reproducible and stable product recovery via foam fractionation could be maintained for 36 h, after a 4 h growth phase.

To summarize, the uncoupled foam fractionation allowed high product enrichment in the foam while biomass concentrations could be reduced for the RL producer and kept low for the HAA producing strain. Even though conditions in the system were permanently changing, e.g., by the actual biomass and surfactant concentrations in the reactor, a constant product removal could be established via uncoupling of the foam fractionation. The outflow of the foam fractionation column is thus superior for subsequent purification steps compared to the reactor discharge. Our setup thus contributes to lowering the operation costs for a potential RL and HAA production process.

### 3.2. Coupled Foam Adsorption Allows In Situ Product Removal

Direct foam adsorption has previously been shown to be a suitable method to handle excessive foaming and serve as a pre-purification step [53]. With the integration of a foam fractionation step before the adsorption, product concentrations are increased, and biomass content is lowered for enhanced adsorption. Furthermore, with a previous fractionation, the quantity of hydrophobic impurities that may also be adsorbed, e.g., pyoverdines, is reduced. In our study, the foam adsorption technique was adopted from Anic et al. [61] and enhanced. The adsorption is based on two alternating adsorption columns, with one column employed for adsorption, while the second column undergoes desorption and regeneration.

**Figure 6:**
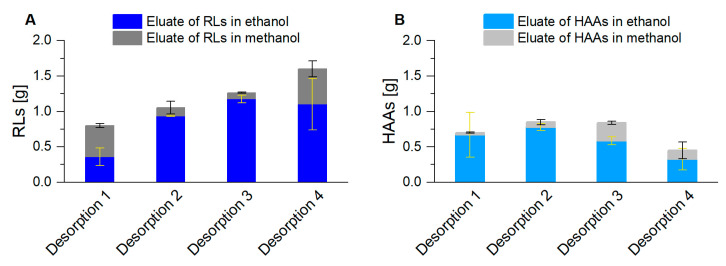
Product harvest ((**A**): RLs; (**B**): HAAs) after foam adsorption and subsequent desorption, first with ethanol (blue) and then with methanol (gray) for each elution. Two adsorption columns alternated every 8 h, for 32 h, resulting in 4 desorption procedures. The error bars indicate the deviation from the mean of two biological replicates.

To configure a continuous and comprehensive packed bed foam adsorption, the specific adsorption capacity for RLs (0.38 g_RL_/g_adsorbent_; [71]) and for HAAs (0.24 g_HAA_/g_adsorbent_; Supplementary Figure S2) was not the only relevant factor, which determined the quantity of applied adsorbent. In addition, e.g., the packed bed dimensions, the exposition time of the surfactant to the adsorbent, channel formations in the packed bed, or impurities on the surface of the adsorbent particles, had to be considered. Samples were taken every 2 h from the permeate, leaving the adsorption column. During the whole harvest phase, no product could be determined in the permeate. With the RL desorption every 8 h, the harvest took place with a constantly increasing product quantity (Figure 6A). In total, 4.7 g RLs were gained in the eluate. For HAA desorption with the same procedure, the highest product quantities were already reached with the second desorption. 0.85 g HAAs could be desorbed, representing the harvest in the cultivation time of 12 h to 20 h (Figure 6B). The sum of all eluted HAAs was 2.8 g.

Consequently, after foam fractionation, a subsequent foam adsorption could be successfully established to separate the surfactants from the remaining culture broth. The product separation avoided process instability by surfactant accumulation in the broth and, therefore, uncontrollable foam formation in the system.

To characterize the efficiency of the whole process, parameters as the final ratio of separated product to the total production, as well as specific yields were determined. The amount of separated biosurfactants was related to the amount of total product synthesized, defined as separation factor *S*_P_ (Equation (8)). For RLs, a final product separation of *S*_RL_ = 40.0% (*w*/*w*) was achieved, with 7.0 g RLs remaining in the reactor. For HAAs, the separation factor of *S*_HAA_ = 99.6% (*w*/*w*) may be interpreted as an effective separation of HAAs from the cultivation process. HAAs are degraded by the bacteria, primarily when lacking dissolved glucose as carbon source, as it could have been the case in the fractionated foam. That is why a share of the product may not have been separated but instead have been degraded. Based on the glucose metabolism, the final yield of product from glucose (Equation (9)), were $Y_{{\mathrm{RL}/S,\;t}_{\mathrm{final}}}=\;{0.07\;g}_{\mathrm{RL}}/g_{S}$, and $Y_{{\mathrm{HAA}/S,\;t}_{\mathrm{final}}}=\;{0.02\;g}_{\mathrm{HAA}}/g_{S}$, values that agree with previous performances [24]. By considering the total cultivation time and the total amount of synthesized product, the STYs (Equation (10)) were ${STY}_{{\mathrm{RL},\;t}_{\mathrm{final}}}=\;{0.16\;g}_{\mathrm{RL}}/L\cdot h$ and ${STY}_{{\mathrm{HAA},\;t}_{\mathrm{final}}}=\;{0.04\;g}_{\mathrm{HAA}\mathrm{HAA}}/L\cdot h$.

### 3.3. Up-Scaling of Fermentation Volume Showcases Robustness of the Presented Process

Industrial production of microbial products is generally achieved by upscaling of the lab-scale process. To achieve similar growth conditions in the up-scaled process, requirements such as sufficient aeration and mixing do not scale linearly with the increased volume but need to be individually adapted, which entails an increased tendency for foam formation [26]. This is often compensated by the usage of chemical defoamers [72]. The suitability of the here developed system for industrial adaptation is thus assessed by increasing the fermentation volume by a factor of 4.5 while maintaining the scale of the downstream setup. In particular, the impact of higher flows into the fractionation column on the fractionation performance as well as on the adsorption efficiency was investigated.

#### 3.3.1. Higher Foam Quantities Decrease the Efficiency of Foam Fractionation

**Figure 7:**
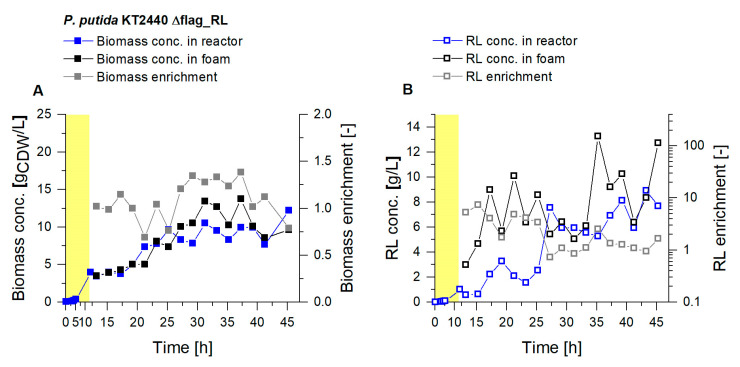
Cultivation of *P. putida* KT2440 Δflag_RL in a bioreactor with 9 L working volume. Growth phase (yellow background) and a subsequent continuous product separation during the harvest phase (t = 12 h to t= 46 h). (**A**) Biomass concentration in the reactor (blue) and in the fractionated foam (black) and the biomass enrichment (*E_X_*, gray). (**B**) Surfactant concentrations were measured in the fermentation broth of the reactor (blue) and in the fractionated foam (black), depicted together with the RL enrichment (*E_P_*, gray).

For the scale-up, a bigger reactor vessel was used, increasing the working volume from 2 L to 9 L. Compared to the smaller culture volume, in the 9 L culture higher biomass concentrations could be obtained (>10 g_CDW_/L) (Figure 7). The RL concentration in the reactor rose constantly and reached values above 8 g_RL_/L, which constitutes a doubling of the concentration compared to the 2 L scale. With an average biomass enrichment in the fractionated foam of 1.1, the biomass enrichment increased by 55% compared to the 2 L scale. In parallel, with an average 2.5-fold RL enrichment in the foam, the product enrichment was significantly lower. The dimensions of the foam fractionation column and the gassing rate for the foam fractionation were not increased with the scale-up. With the 9 L culture volume, the higher RL concentrations in the broth caused a foam with a higher liquid content, which could not be sufficiently fractionated in the applied fractionation column. The foam that left the upper outlet of the column still contained larger shares of liquid, with less enriched product fractions. Consequently, an enlargement of the column dimensions is necessary to achieve similar product concentrations and enrichments on a 9 L scale as in 2 L cultures.

**Figure 8:**
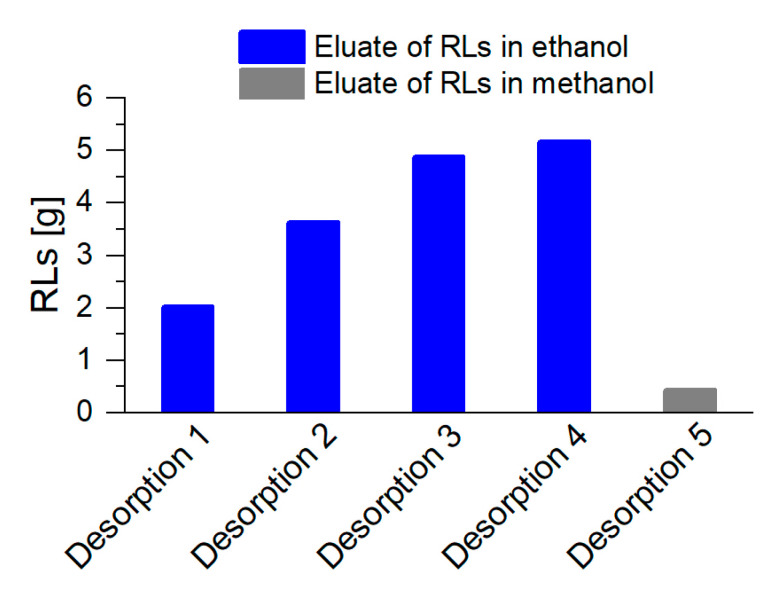
RL harvest after foam adsorption and a subsequent desorption, with ethanol as eluent for the regular elution during cultivation (blue, desorption 1 to 4). Two adsorption columns alternated every 8.5 h, for 34 h, resulting in 4 desorption procedures. Final desorption with methanol was executed at the end of the cultivation (gray, desorption 5).

By the desorption of the RLs every 8.5 h during the harvest phase, an increasing product quantity could be collected from 2 g RLs for the first, and up to 5 g RLs for the fourth desorption (Figure 8). With a final rinse of the columns with methanol, only a minor product amount was eluted, indicating that the major quantity of RLs was already desorbed previously. In total, 16 g RLs were separated from the cultivation broth via integrated foam fractionation and adsorption, while 69 g RLs remained in the reactor. This resulted in a final product separation of *S*_RL_= 19% (*w*/*w*). With ${STY}_{{\mathrm{RL},\;t}_{\mathrm{final}},\;9L}=\;{0.21\;g}_{\mathrm{RL}}/L\cdot h$, the STY increased by 31% with a 4.5-fold increased culture volume. Intriguingly, even with an increased culture volume and thus significantly high foam formation, no chemical defoamers were necessary to maintain process stability over the whole cultivation time. Beuker et al. [36] reached an equal STY as determined in our work, only by the heavy use of chemical defoamers, with a final product titer of ${15\;g}_{\mathrm{RL}}/L$. Without this defoamer, the subsequent downstream processing is simplified, and production costs are generally lower.

While the STY could be increased with a system scale-up, the efficiency of the fractionation column declined, e.g., indicated by a drop of the separation factor to half the value reached at the 2 L scale. With the 9 L culture volume, the amount of accumulated RLs has exceeded the capacity of the applied foam fractionation. Fewer RLs could be separated by adsorption onto the gas-liquid interface than being secreted by the whole-cell biocatalysts into the culture broth.

#### 3.3.2. Fractionated Foam Causes Steady Permeate Flow through the Adsorption Column

**Figure 9:**
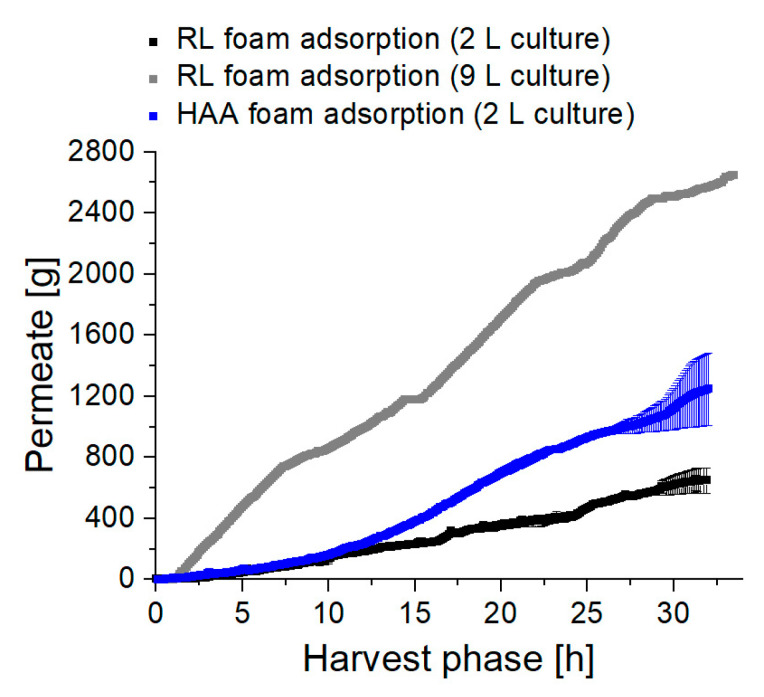
Weight of permeate collected after the adsorption column over the course of the harvest phase. Permeate trends in a 2 L working volume for the RL synthesis (black), and HAA synthesis (blue). The error bars indicate the deviation from the mean of two biological replicates. The permeate trend for RL synthesis in a 9 L working volume is plotted in gray.

The impact of higher flows on the performance of the integrated foam adsorption was assessed. By tracking the weight of the collected liquid flowing out of the adsorption column, i.e., the permeate, the liquid quantities separated from the cultivation with the fractionated foam could be determined (Figure 9). For RL synthesis in the 2 L culture volume, a final permeate weight of 0.65 kg was measured. With our previously performed integrated foam fractionation [20], which was highly dependent on the bioreactor process parameters, 610 mL foamate were separated with 2.5 g RLs. With the new foam fractionation column design and the subsequent foam adsorption presented in this study, 649 mL permeate were separated, and 4.7 g RLs could be obtained after desorption. Concluding, with a slightly increased quantity of separated liquid, the mass of product separated with the fractionated foam could be increased 1.9-fold.

During HAA production with 1.25 kg, about twice the mass of foamed liquid was separated compared to RL production. As a generally higher foaming capability for RLs was determined previously [20,47], it is assumed that other components in the culture broth promote foam formation. Mainly cells, lysed cells, and secreted proteins are known to increase the foaming of a culture broth [34,73]. This statement is supported by the observed HAA-free foam after the adsorption column, containing the same biomass concentration as the foam that is entering the adsorption column. Obviously, cells did not agglomerate in the packed bed of the adsorbent but may have promoted foaming at biomass concentrations above 14 g_CDW_/L, which were much higher biomass concentrations as measured in the foam for RL synthesis.

With the 9 L working volume for RL synthesis, after 32 h of the harvest phase, 2.6 kg permeate was collected. While the working volume was scaled-up by factor 4.5, foaming increases by a factor of 4. The pressure in the adsorption column generally increased from <0.8 bar in the 2 L scale to >1.2 bar in the 9 L scale. This pressure build-up must be considered when evaluating the permeate flow, as foaming, and therefore the transfer of interstitial liquid is reduced by increasing back-pressure [72]. However, the 4.5-fold scale-up of the working volume led to a 7.2-fold increased total RL production, from $m_{\mathrm{RL},\;2\;L}=\;12\;g$ to $m_{\mathrm{RL},\;9\;L}=\;85\;g$, revealing the high potential of system scale-up for enhanced RL and HAA production.

## 4. Discussion

### 4.1. Combining Previous Knowledge with Recent Findings Enabled the Design of a Highly Efficient System for Biosurfactant Production

We here present a biosurfactant production and recovery system, operating without pathogenic strains and chemical defoamers. Siemann et al. [59] already described a workflow with a product adsorption onto a hydrophobic resin after the RLs were separated from the discharged and collapsed foam by precipitation. With the development of a foam adsorption technique [61,63], allowing a direct load of the adsorption column with foam discharged from the cultivation process, the system was simplified and became more efficient. Furthermore, to prevent bacterial foam adhesion, Siemann et al. [59] immobilized the applied RL producer *P. aeruginosa* DSM 2874 in an individually designed fluidized bed reactor. This system, e.g., lacking of an insufficient oxygen supply, could be improved by Heyd et al. [62], capturing the same strain in magnetic alginate beads. The bacterial cells could be retained by guiding the discharged foam from an aerated and stirred bioreactor through a magnetic field. Even though the system could be operated under stable conditions for three weeks, the STY did not exceed ${0.023\;g}_{\mathrm{RL}}/L\cdot h$. In the following, Beuker et al. [60] optimized the process conditions for suspended cells of *P. putida* KT2440 pSynPro8oT_*rhlAB* in a bioreactor, reaching higher STYs than the previous setups with immobilized cells. RLs were more than 10-fold enriched during the whole process, which are higher enrichments as were achieved in the present study, but the total quantity of produced RLs was rather low. Anic et al. [61] produced 16 g RLs and therefore about 16 times more RLs than Beuker et al. [60], using *P. putida* EM383 pPS05_*rhlAB*, by applying a foam adsorption to separate the RLs and to recycle the culture broth that was entrapped in the foam. We did not recycle the discharged liquid, i.e., the permeate, to enable a constant reactor working volume, as glucose solution and medium were fed during the harvest phase. By applying the upstream foam fractionation, the products could already be separated from a larger share of liquid and biomass. Consequently, the accumulation of impurities and clogging of the packed bed adsorbent by entrained culture broth was reduced while higher RL enrichments in the foam could be realized. The higher product concentrations in the foam were reached at the expense of a lower product separation of 40% as larger product shares could be detected in the drained liquid, while Anic et al. [61] reported a complete product separation. An inversely proportional dependence of mono-RL recovery and enrichment was also shown when different gassing rates were investigated for foam fractionation [57].

Compared to our previous cultivations of the applied RL and HAA producing strains with integrated foam fractionations without subsequent adsorption [24], the STYs have fallen by 24% and 43%, respectively. The higher yields in the earlier experiments were certainly achieved by initiating the harvest phase after reaching a biomass concentration of 5 g_CDW_/L in the 2 L culture volume (in this study already at 1 g_CDW_/L), as generally larger quantities of biomass form more product. However, compared to similar bioprocesses performed by Beuker et al. [60] and Anic et al. [61], the STY for RL production in our work was 4.2-fold and 2.2-fold higher, respectively. Higher STYs were reported only by applying chemical defoamers, reaching about ${0.2\;g}_{\mathrm{RL}}/L\cdot h$ [36]. However, we could achieve an equal STY without chemical defoamers by a 4.5-fold system scale-up, leading to 16 g of separated RLs. For HAAs, the reduced STY compared to previous cultivations [24] might be traced back again to the molecules’ degradability, promoted by longer residence times given by larger column dimensions, and higher cell densities in the foam. Primarily because of their instability, HAAs had to be recovered from the ongoing cultivation process. For RL synthesis, a 1.4-fold higher separation factor could be achieved, compared to the previously applied foam fractionation, which was dependent on bioreactor process parameters [24]. In addition, the separated surfactants were recovered in higher purity due to subsequent adsorption and desorption.

Foam fractionation in an external column that is integrated into a bioreactor process, has already been beneficial to produce the hydrophobin protein HFBII. The culture broth was continuously pumped into the column, achieving product recoveries of 70% with a minor product loss by foam overflow through the gas exhaust [56]. As we used here discharged foam instead of fermentation broth for product separation, product loss by foam overflow could be prevented.

Since the patenting of the foam adsorption technique for RL recovery in 2013 [63], published processes defined the loss of biocatalysts by bacterial foam adhesion, and an associated pressure build-up in the adsorption column as a major concern for process stability [61,71,74]. The adsorbent C_18_ silica-based ODS-A, as used in our work, was reported previously as most suitable for an integrated RL foam adsorption [61,71]. Furthermore, we avoid a transfer of the pressure build-up in the adsorption column to the upstream fractionation column by a peristaltic pump in between these columns. With a 9 L culture volume, the higher pressure in the adsorption column also impacted the pressure in the fractionation column. With a further scale-up, the increased column pressure may exceed the maximum discharge pressure of a usual peristaltic pump (<10 bar). A packed-bed adsorption column design offers only a few possibilities to avoid pressure build-up, as a larger column diameter at a constant bed volume or larger adsorbent particles promote channel formation. In conclusion, most efficient pressure avoidance is most likely achieved by frequent alternating column use, allowing a column regeneration in shorter time intervals. Much more potential for technical improvement is given by the upstream foam fractionation, leading to a reduced load of the adsorption column with culture broth.

### 4.2. Fermentation Products Impact Foam Formation and Stabilization

Primarily during the production of HAAs, foam formation and stabilization was not exclusively caused by the HAAs itself. In general, secreted, or by cell autolysis released proteins and amino acids, are the most common surface-active substances in bioprocesses [73,75]. It is assumed that cell lysis has a self-reinforcing effect on foam formation, e.g., by stressed cells that are entrapped in the foam and thus also tend to lyse [33]. Several studies describe microbially secreted proteins that are separated by foam fractionation, like nisin, lipases, and cutinases [76,77,78,79]. Davis et al. [80] observed in a bioprocess with integrated foam fractionation for surfactin recovery, that protein concentrations in the foam were at least five times higher than the concentration of surfactin.

Microorganisms themselves benefit from a specific cell surface hydrophobicity (CSH) [81], e.g., the hydrophobic cell surface of pseudomonads favors solvent tolerance [82,83]. Own studies revealed that certain bacterial surface structures of *P. putida* KT2440, and especially the flagellum promote bacterial foam adhesion [24]. The here applied HAA producing strain still has the flagellum while it was deleted for the RL producer. This might explain why biomass enrichment in the foam was 1.6-fold higher for HAA production. By the adhesion of bacterial cells onto the gas-liquid interface of the foam, the foam may also be stabilized. In particular for early-stage RL production by *P. aeruginosa*, the bacteria itself was defined as primary cause for foaming [34]. Davis et al. [80] describe an increased foam formation in a culture broth of *Bacillus subtilis*, compared to a cell-free system. Discussions whether bacteria contribute to foam formation or merely stabilize the existing foam are versatile [84]. Therefore, the impact of the microorganism and the culture itself on the foam fractionation performance must be considered right from the early stage of the bioprocess development.

### 4.3. Further Technical Enhancements in Foam Fractionation Are Foreseeable

With the applied fractionation column, the product could be separated from the cultivation process with a 6.3-fold RL enrichment and a 17-fold HAA enrichment. However, the largest share of product that is introduced into the fractionation column is pumped back into the reactor via the drainage reflux pump. Only 8% of RLs and 22% of HAAs were recovered. For a more efficient system scale-up, larger column dimensions would be feasible to increase the residence time of the foam in the column and, therefore, the time for the interstitial liquid to drain [31]. Furthermore, a foam fractionation column allows versatile designs for optimized product recovery. The gas introduction into the liquid pool, and therefore the size and quantity of gas bubbles is controlled by the individual sparger and gassing rate [30,85], e.g., with smaller pores in the sparger, at a constant gassing rate, smaller gassing bubbles are introduced into the liquid, leading to an increased gas-liquid interface. By using a sparger with smaller pores, Sarachat et al. [30] obtained higher RL recoveries at a constant gassing rate. Fractionation column internals provide a larger surface area, on which foam drainage can occur. Compared to a conventional foam fractionation column, Dickinson et al. [86] achieved a 4-fold higher enrichment of cetyltrimethylammonium bromide (CTAB) by applying parallel inclined channels for fractionation. By integrating a spiral into the fractionation column, Yang et al. [87] reached a 2.5-fold enrichment of sodium dodecyl sulfate (SDS) in the fractionated foam. In another approach, using a wire gauze structured packing, a 2.4-fold higher enrichment of bovine serum albumin (BSA) was reported by Li et al. [88]. Other studies describe the use of multiple stages for an enhanced foam fractionation. For example, Darton et al. [89] injected air through a sparger at each stage for the purification of octylphenol polyethoxylate (Triton X-100), and cetylpyridinium chloride (CPC). With this technique, 10-fold and 5-fold enrichments of Triton X-100 and CPC were reached, respectively. Boonyasuwat et al. [90] and Rujirawanich et al. [91] used specific perforated trays separating the column into sections to collect the drained liquid for a higher enrichment and recovery of CPC. By the sudden de- and increasing of flow areas, as achieved by separating the column with a plate that only allowed the rise of the foam through a narrower tube, the liquid flux of an SDS foam could be reduced by 35% [92]. For continuous foam fractionation, stripping or enriching modes are most common, differing in the position of the inlet of the dissolved surfactants in the fractionation column [78]. In a stripping mode, the surfactant solution is introduced directly into the rising foam, where the surfactant adsorption mainly takes place. For RLs, product recoveries of 96% were already achieved in a stripping mode, using a fractionation column that is not integrated into the bioreactor process [57]. Thus, an increased recovery of RLs and HAAs is also expected when operating an integrated foam fractionation column in stripping mode. Maybe an increased recovery is already achieved when culture broth or the drained liquid from the external fractionation column is sprinkled into the foam of the bioreactor headspace. Future projects should investigate these aspects for more efficient RL and HAA foam fractionation, enabling production with integrated product recovery.

## 5. Conclusions

The shown reactor setup allows product separation via foam fractionation that is performed independently from bioreactor operation, especially relevant for a system scale-up, enabling individual reactor and fractionation column designs.

By carrying out a three-step operation, initiated by the cultivation, followed by a foam fractionation for liquid and biomass removal, and a final product adsorption and desorption, a continuous RL and HAA production and purification process could be established. A true understanding of physical parameters, but also of the influence of cell surface properties, especially of the fractionation column, will help to further optimize RL and HAA production.

## 6. Patents

L.M.B. and T.T. declare that they are inventors of three related patents. (1) L. M. Blank, F. Rosenau, S. Wilhelm, A. Wittgens, T. Tiso, “Means and methods for RL production” HHU Düsseldorf University, TU Dortmund University, 2013 (WO 2013/041670 A1), (2) L. M. Blank, B. Küpper, E. M. del Amor Villa, R. Wichmann, C. Nowacki, “Foam adsorption” TU Dortmund University, 2013 (WO 2013/087674 A1), and (3) L. M. Blank, T. Tiso, A. Germer, “Extracellular production of designer hydroxyalkanoyloxy alkanoic acids with recombinant bacteria” RWTH Aachen University, 2015 (WO2017006252A1).

## Figures and Tables

**Table 1:** Fermentative RL production and separation via integrated and non-integrated foam fractionation systems.

Organism	System ^1^	Medium Volume [L]	Carbon Source	Space-Time Yield [g_RL_/L·h]	Produced RLs [g_RL_]	Reference
*P. aeruginosa* DSM 2874	I	18	glycerol	0.043	16	[59]
*P. aeruginosa* DSM 2874	I	6	glycerol	0.023	70	[62]
*P. putida* KT2440 ^2^	S	1.5	glucose	0.038	1	[60]
*P. putida* EM383 ^2^	S	2.5	glucose	0.073	16	[61]
*P. putida* KT2440 ^3^	S	2	glucose	0.24	10	[24]

^1^ S: Suspended cells and I: Immobilized cells; ^2^ Plasmid based and ^3^ genome integrated production genes (*rhlAB*).

## Supplementary Materials

The following are available online at https://www.mdpi.com/2076-2607/8/12/2029/s1, Figure S1: Picture of fermentation setup. Figure S2: Determination of the maximum HAA adsorption capacity with Octadecylsilyl-A AA12SA5 as adsorbent. Table S1: Bioreactor and foam fractionation process parameters at a 2 L bioreactor working volume. Table S2: Alternating adsorption, desorption and regeneration procedure for the adsorption columns with a packed bed of 30 g C18 silica-based ODS-A, for continuous product separation at a 2 L bioreactor working volume. Table S3: Bioreactor and foam fractionation process parameters at a 9 L bioreactor working volume that deviate from the bioreactor process in a 2 L working volume. Table S4: Alternating adsorption-, desorption- and regeneration procedure for the adsorption columns with a packed bed of 60 g C18 silica-based ODS-A, for continuous product separation at a 9 L bioreactor working volume.
