# Supplementary material for: Uncoupling Foam Fractionation and Foam Adsorption for Enhanced Biosurfactant Synthesis and Recovery
Source: Microorganisms. 2020 Dec 18;8(12):2029. doi: 10.3390/microorganisms8122029 (PMC7766737; doi:10.3390/microorganisms8122029)
Supplement: Supplementary file 1 [file microorganisms-08-02029-s001.pdf]

microorganisms

Supplementary Material

Uncoupling Foam Fractionation and Foam Adsorption for Enhanced Biosurfactant Synthesis and Recovery

Christian C. Blesken [1], Tessa Strümpfler [1], Till Tiso [1,*] and Lars M. Blank [1,*]

[1] iAMB - Institute of Applied Microbiology, ABBt - Aachen Biology and Biotechnology, RWTH Aachen University, Aachen, Germany

* Correspondence: till.tiso@rwth-aachen.de & lars.blank@rwth-aachen.de; Tel.: +49-241-8026601

Received: date; Accepted: date; Published: 18 December 2020

1. Supplementary tables

1.1. Process parameters of the fermentation setup and procedure

Table S1. Bioreactor and foam fractionation process parameters at a 2 L bioreactor working volume

Process parameter	Value
Total vessel volume	3 L
Stirring speed	500 rpm
Temperature	30°C
Reactor sparger pore size	20 µm
Reactor sparger surface area	19.4 cm²
Foam centrifuge rotation speed	4000 rpm
Pump rate of foamate reflux	265 mL/min
Pump rate of drainage reflux	17 mL/min
Fractionation column sparger pore size	20 µm
Fractionation column sparger surface area	7.1 cm²
Added 50% (w/v) glucose sol. per feed pulse	14 g (*i.e.*, 6 g glucose)

Table S2. Alternating adsorption, desorption and regeneration procedure for the adsorption columns with a packed bed of 30 g C_{18} silica-based ODS-A, for continuous product separation at a 2 L bioreactor working volume.

Column status	Duration	Volume flow	$V_{pumped\ liquid}/V_{adsorbent}$
Adsorption	8 h		
Blow with air [1]	2 min	0.5 L/min	
Flush with water	10 min	10 mL/min	1.8
Blow with air [1]	2 min	0.5 L/min	
Desorption with ethanol	1 h	8 mL/min	8.5
Blow with air [1]	2 min	0.5 L/min	
Desorption with methanol	1 h	8 mL/min	8.5
Blow with air [1]	2 min	0.5 L/min	
Flush with water	10 min	10 mL/min	1.8
Blow with air [1]	2 min	0.5 L/min	

[1]) At 0.5 bar overpressure

Table S3. Bioreactor and foam fractionation process parameters at a 9 L bioreactor working volume that deviate from the bioreactor process in a 2 L working volume.

**Process parameter**	**Value/ type**
Total vessel volume	13 L
Reactor sparger	Ring sparger [1]
Added 50% (w/v) glucose sol. per feed pulse	90 g (*i.e.*, 38 g glucose)

[1]) As provided for this vessel by Eppendorf AG, Hamburg, Germany

Table S4. Alternating adsorption-, desorption- and regeneration procedure for the adsorption columns with a packed bed of 60 g C_{18} silica-based ODS-A, for continuous product separation at a 9 L bioreactor working volume.

**Column status**	**Duration**	**Volume flow**	$V_{\text{pumped liquid}}/V_{\text{adsorbent}}$
Adsorption	8 h		
Blow with air [1]	2 min	0.5 L/min	
Flush with water	10 min	10 mL/min	0.9
Blow with air [1]	2 min	0.5 L/min	
Desorption with ethanol	2 h	8 mL/min	8.5
Blow with air [1]	2 min	0.5 L/min	
Flush with water	10 min	10 mL/min	0.9
Blow with air [1]	2 min	0.5 L/min	
Final desorption step after 2nd desorption with ethanol			
Desorption with methanol	30 min	8 mL/min	2.1
Blow with air [1]	2 min	0.5 L/min	

[1]) At 0.5 bar overpressure

2. Supplementary figures

2.1. Supplementary figures

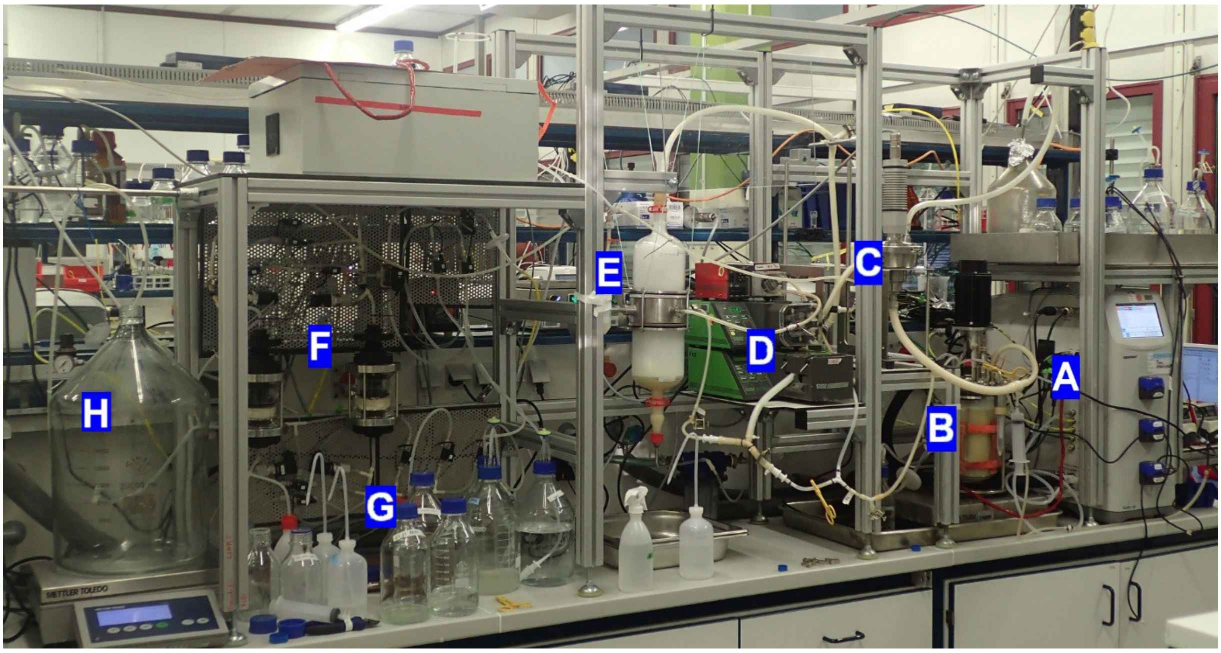

Figure S1. Picture of fermentation setup. The BioFlo 120 bioreactor system, including a control unit (A) and the bioreactor itself (B), was applied for microbial biosurfactant synthesis. The foam was discharged through the reactor exhaust to a foam centrifuge (C) to separate offgas and foamate. The foamate was pumped (D) into the foam fractionation column (E). The fractionated foam was led to an automated adsorption unit (F). Non-adsorbed substances were collected as permeate (H). At product desorption, the eluate was collected separately (G).

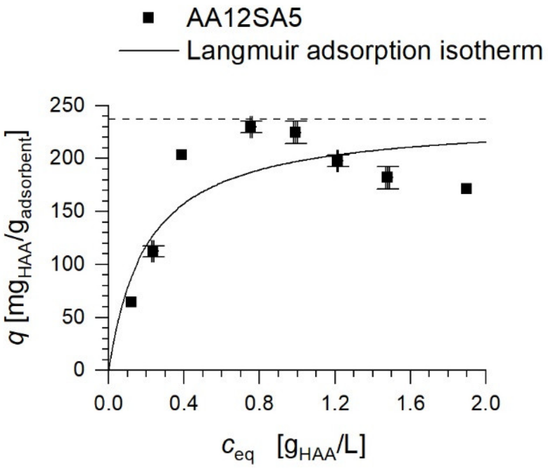

Figure S2. Determination of the maximum HAA adsorption capacity with Octadecylsilyl-A AA12SA5 as adsorbent. Adsorbed HAA per quantity of adsorbent (q, black squares) vs. HAA concentration in the supernatant (c_{eq}). Langmuir adsorption isotherm fit (black line) with a maximum adsorption capacity of q_{max}= 237 $mg_{HAA}/g_{adsorbent}$ (dashed line). The error bars indicate the deviation from the mean of two replicates.

© 2020 by the authors. Licensee MDPI, Basel, Switzerland. This article is an open access article distributed under the terms and conditions of the Creative Commons Attribution (CC BY) license (http://creativecommons.org/licenses/by/4.0/).
